# The Inertial Attitude Augmentation for Ambiguity Resolution in SF/SE-GNSS Attitude Determination

**DOI:** 10.3390/s140711395

**Published:** 2014-06-26

**Authors:** Jiancheng Zhu, Xiaoping Hu, Jingyu Zhang, Tao Li, Jinling Wang, Meiping Wu

**Affiliations:** 1 Department of Automatic Control, College of Mechatronics and Automation, National University of Defense Technology, Deya Street 109, Changsha 410073, Hunan, China; E-Mails: Xphu@nudt.edu.cn (X.H.); litao_nudt@yahoo.com.cn (T.L.); meipingwu@263.net (M.W.); 2 School of Civil and Environmental Engineering, University of New South Wales, Sydney, NSW 2052, Australia; E-Mail: Jinling.Wang@unsw.edu.au

**Keywords:** GNSS attitude determination, SF/SE-GNSS compass, inertial attitude augmentation, IDBV, MEMS-IMU, ADOP, integer aperture estimation

## Abstract

The Unaided Single Frequency/Single Epoch Global Navigation Satellite System (SF/SE GNSS) model is the most challenging scenario for ambiguity resolution in the GNSS attitude determination application. To improve the performance of SF/SE-GNSS ambiguity resolution without excessive cost, the Micro-Electro-Mechanical System Inertial Measurement Unit (MEMS-IMU) is a proper choice for the auxiliary sensor that carries out the inertial attitude augmentation. Firstly, based on the SF/SE-GNSS compass model, the Inertial Derived Baseline Vector (IDBV) is defined to connect the MEMS-IMU attitude measurement with the SF/SE-GNSS ambiguity search space, and the mechanism of inertial attitude augmentation is revealed from the perspective of geometry. Then, through the quantitative description of model strength by Ambiguity Dilution of Precision (ADOP), two ADOPs are specified for the unaided SF/SE-GNSS compass model and its inertial attitude augmentation counterparts, respectively, and a sufficient condition is proposed for augmenting the SF/SE-GNSS model strength with inertial attitude measurement. Finally, in the framework of an integer aperture estimator with fixed failure rate, the performance of SF/SE-GNSS ambiguity resolution with inertial attitude augmentation is analyzed when the model strength is varying from strong to weak. The simulation results show that, in the SF/SE-GNSS attitude determination application, MEMS-IMU can satisfy the requirements of ambiguity resolution with inertial attitude augmentation.

## 1. Introduction

The weak Global Navigation Satellite System (GNSS) model is defined as a scenario with insufficient observation quantity or poor measurement quality, so that the availability of the GNSS application becomes worse. As to the GNSS attitude determination, weak GNSS model strength can be caused by few available GNSS satellites and frequencies, insufficient epochs and noisy measurements, *etc.* [1–5]. These factors lead to the failure of reliable but unaided ambiguity resolution. The increasing number of urban canyon environments makes satellites’ signals easily blocked or contaminated by multipath signals. The highly dynamic movement of vehicles also indirectly weakens the model strength [6]. In these situations, it is difficult to realize the ambiguity resolution of the GNSS attitude determination system based on low-end receivers.

Unaided SF/SE-GNSS ambiguity resolution is a typical GNSS application in a weak GNSS model. The augmentation methods can be categorized into three classes: multi-GNSS, multi-sensor information fusion and *a priori* constraints. Multi-GNSS can increase the data redundancy and improve the system reliability [7]. Multi-sensor information fusion, or integrated navigation, can overcome the shortcomings of different sensors. The vehicle attitude, baseline vector or difference carrier phase measurements are usually input to the optimal estimator for improving the availability of GNSS applications with weak model strength [8,9]. Typically, *a priori* constraints include the baseline length [10], the geometry of multi-baselines [11] and the non-holonomic constraints [12]. All these constraints can be used to decrease the ambiguity search space and improve the success rate of ambiguity resolution.

From the 1990s on, methods of inertial measurement augmentation for ambiguity resolution appeared successively in both studies on GNSS attitude determination and Strapdown Inertial Navigation System/GNSS (SINS/GNSS) integrated attitude determination. In 1998, to improve the accuracy of GNSS attitude determination, Hayward and Egziabher made use of gyro measurements to smooth the high frequency errors contained in GNSS carrier phase measurements [13,14]. In 1999, Han showed that the inertial attitude measurement from a rate-gyro can be used to improve the success rate of ambiguity resolution [15]. From 2001 to 2003, Yang revealed that the MEMS-IMU measurement can be used as an *a priori* constraint to improve the integrity of ambiguity resolution [9,16]. In 2004, to resolve the problems existing in the GNSS attitude determination application when low-end receivers are used, Wang introduced the gyro-rate measurement into the attitude integration filtering and improved the performance of GNSS attitude determination system [17,18]. In 2008, Dickman studied how to use the inertial measurement to depress the low frequency errors contained in GNSS carrier phase measurements [19], but this method requires a high IMU measurement precision, hence, it is not applicable to GNSS attitude determination.

In theory, the method of inertial attitude augmentation for ambiguity resolution is not the same as that of the multi-sensor information fusion augmentation. Traditional SINS/GNSS integrated navigation systems use the SINS measurement error as the state vector, and the GNSS output as the observations. However, the error characteristics of low-end IMU measurements vary greatly with the vehicle dynamics and working temperature. In a weak GNSS model, it is very difficult to achieve the optimal estimation for SINS errors and the filter divergence will lead to the unavailability of the GNSS application. As the output frequency of the low-end receivers gradually increases, the GNSS attitude determination system can satisfy the frequency requirements in common dynamic situations. The MEMS-IMU measurement within a short span can be used as a high quality prior constraint to augment the GNSS model strength.

In this paper, Section 1 reviewed the development of inertial augmentation for the GNSS model and introduces the meanings of this research. Section 2 will give the definition of IDBV and generate an inertial augmentation ambiguity search space based on the IDBV and SF/SE-GNSS compass. Then, the geometry characteristics of this search space are also analyzed. Section 3 will reveal the mechanism of inertial attitude augmentation for SF/SE-GNSS model strength. The ADOP is introduced and utilized to analyze the effect of inertial attitude measurement for SF/SE-GNSS model strength augmentation. Section 4 will analyze and compare the performance of ambiguity resolution based on an integer aperture estimator with fixed failure rate after simulation experiments. Finally, Section 5 will provide a summary of the work.

## 2. Inertial Attitude Aided Ambiguity Search Space

This section gives the definition of IDBV, and then generates an ambiguity search space based on a float ambiguity vector and its Variance-Covariance Matrix (VCM). The obtained space is called the inertial attitude aided ambiguity search space. By analyzing the geometry characteristics of this space, how the inertial attitude measurement imposes its influence to the unaided SF/SE-GNSS ambiguity search space will be clarified.

### 2.1. IDBV

In a certain GNSS epoch, the attitude measurement of SINS is denoted as ${\text{C}}_{\text{b}}^{\text{n}}$, thus, the IDBV can be defined as:

(1) $${\text{b}}_{\text{I}}^{\text{n}}={\text{C}}_{\text{b}}^{\text{n}}{\bar{\text{b}}}^{\text{b}}$$

In Equation (1), ${\bar{\text{b}}}^{\text{b}}$ denotes the true value of the baseline vector in the body-frame (b-frame). Then, *δ*(·) is introduced to represent the deviation between the measurement and true value. The following equation is thus obtained [20]:

(2) $$\text{δ}{\text{b}}^{\text{n}}=-\left({\text{γ}}^{\text{n}}\times \right)\cdot {\bar{\text{b}}}^{\text{n}}$$

In Equation (2), ${\text{γ}}^{\text{n}}={\left[\gamma_{x}^{\text{n}},\gamma_{y}^{\text{n}},\gamma_{z}^{\text{n}}\right]}^{T}$ is to represent the rotational angle error of ${\text{C}}_{\text{b}}^{\text{n}}$ in navigation frame (n-frame). Equation (2) establishes the relation between the measurement errors of inertial attitude and IDBV.

The differential equation of **γ**^n^ with respect to time can be expressed as [21]:

(3) $${\dot{\text{γ}}}^{\text{n}}=-{\bar{\text{C}}}_{\text{b}}^{\text{n}}\text{δ}{\text{ω}}_{\text{ib}}^{\text{b}}-\left({\bar{\text{ω}}}_{\text{in}}^{\text{n}}\times \right){\text{γ}}^{\text{n}}+\text{δ}{\text{ω}}_{\text{in}}^{\text{n}}$$

In Equation (3), $\text{δ}{\text{ω}}_{\text{ib}}^{\text{b}}$ is the measurement error vector of the gyros, and if MEMS gyros with the current accuracy level are utilized, each component of $\text{δ}{\text{ω}}_{\text{ib}}^{\text{b}}$ has an order of magnitude of 30 ~ 200deg/h (≈1.5×10^−1^ ~ 1mrad/s). ${\bar{\text{ω}}}_{\text{in}}^{\text{n}}$ denotes the true value of the n-frame angular velocity vector with respect to the inertial frame (i-frame) and represented in n-frame. For a common land vehicle maneuvering on the Earth’s surface, e.g., the maximum linear velocity is limited to 200 km/h, each component of ${\bar{\text{ω}}}_{\text{in}}^{\text{n}}$ will have the same order of magnitude as the angular rate of the Earth’s rotation, *i.e.*, Ω = 7.292115×10^−2^. After a cross product with **γ**^n^, each component of which is assumed to be less than 0.01deg (≈1.7×10^−1^mrad), the second item on the right side of Equation (3) will have a 1.2×10^−2^ mrad/s order of magnitude. $\text{δ}{\text{ω}}_{\text{in}}^{\text{n}}$ denotes the measurement error contained in the measurement of ${\bar{\text{ω}}}_{\text{in}}^{\text{n}}$. If the time intervals between different GNSS observations are very short, and GNSS position and velocity are both available with accuracy levels of 10 m and 0.1 m/s, respectively, and it can be proved that each component of $\text{δ}{\text{ω}}_{\text{in}}^{\text{n}}$ has a 10^−4^mrad/s order of magnitude. Hence, Equation (3) can be approximated as:

(4) $${\dot{\text{γ}}}^{\text{n}}\approx -{\text{C}}_{\text{b}}^{\text{n}}\text{δ}{\text{ω}}_{\text{ib}}^{\text{b}}$$

It can be noted that Equation (4) omits the influence of vehicle dynamic. Hence, in this situation, the measurement error of the gyros is the principal contributor to the divergence speed of the inertial attitude measurement error.

We denote the neighboring two epochs as *t*_0_ and *t*_1_, *t*_0_ is the starting time of the SINS calculation. The initial attitude at *t*_0_ is provided by a GNSS attitude determination system with two orthogonal but equal-length baselines. The inertial attitude will be the integral of the MEMS-IMU measurements and starting from the initial attitude. With Equation (4), the inertial attitude measurement error at *t*_1_ equals:

(5) $${\text{γ}}^{\text{n}}\left(t_{1}\right)={\text{γ}}^{\text{n}}\left(t_{0}\right)-\int_{t_{0}}^{t_{1}}{\text{C}}_{\text{b}}^{\text{n}}\left(t\right)\text{δ}{\text{ω}}_{\text{ib}}^{\text{b}}\left(t\right)dt$$

The common model of $\text{δ}{\text{ω}}_{\text{ib}}^{\text{b}}$ is [21,22]:

(6) $$\text{δ}{\text{ω}}_{\text{ib}}^{\text{b}}={{\text{K}}_{\text{Bias}}^{\text{b}}}^{\prime }+{w_{\text{Rand}}^{\text{b}}}^{\prime }$$

where ${{\text{K}}_{\text{Bias}}^{\text{b}}}^{\prime }$ is the equivalent gyro bias, which is usually modeled as a random constant vector. The equivalent measurement noise ${{\text{w}}_{\text{Rand}}^{\text{b}}}^{\prime }$ is modeled as a white noise vector. Substituting Equation (6) into Equation (5), then it is obtained that:

(7) $$\begin{array}{ll} {\text{γ}}^{\text{n}}\left(t_{1}\right) & ={\text{γ}}^{\text{n}}\left(t_{0}\right)-\int_{t_{0}}^{t_{1}}{\text{C}}_{\text{b}}^{\text{n}}\left(t\right)\left({{\text{K}}_{\text{Bias}}^{\text{b}}}^{\prime }\right)dt-\int_{t_{0}}^{t_{1}}{\text{C}}_{\text{b}}^{\text{n}}\left(t\right)\left({{\text{w}}_{\text{Rand}}^{\text{b}}}^{\prime }\right)dt\\ & ={\text{γ}}^{\text{n}}\left(t_{0}\right)-\int_{t_{0}}^{t_{1}}{\text{C}}_{\text{b}}^{\text{n}}\left(t\right)\left({{\text{K}}_{\text{Bias}}^{\text{b}}}^{\prime }\right)dt-\int_{t_{0}}^{t_{1}}\left({{\text{w}}_{\text{Rand}}^{\text{n}}}^{\prime }\right)dt\\ & ={\text{γ}}^{\text{n}}\left(t_{0}\right)-\int_{t_{0}}^{t_{1}}{\text{C}}_{\text{b}}^{\text{n}}\left(t\right)\left({{\text{K}}_{\text{Bias}}^{\text{b}}}^{\prime }\right)dt-{\text{ν}}_{\text{Walk}}^{\text{n}} \end{array}$$

${\text{C}}_{\text{b}}^{\text{n}}$ is an orthogonal matrix, hence, ${{\text{w}}_{\text{Rand}}^{\text{n}}}^{\prime }={\text{C}}_{\text{b}}^{\text{n}}\left(t\right)\left({{\text{w}}_{\text{Rand}}^{\text{b}}}^{\prime }\right)$ retains the statistical characteristics of ${\text{w}}_{\text{Rand}}^{\text{b}}$ [12,23], which means ${{\text{w}}_{\text{Rand}}^{\text{n}}}^{\prime }$ is still a white noise vector. A random walking item ${\text{ν}}_{\text{Walk}}^{\text{n}}$ will be obtained by integrating the white noise vector ${{\text{w}}_{\text{Rand}}^{\text{n}}}^{\prime }$ with respect to *t*. On the other hand, ${\text{C}}_{\text{b}}^{\text{n}}\left(t\right)$ is continuous in closed interval [*t*_0_, *t*_1_] and integrable in open interval (*t*_0_, *t*_1_). According to the first integral mean theorem, there is:

(8) $$\begin{array}{ll} \int_{t_{0}}^{t_{1}}{\text{C}}_{\text{b}}^{\text{n}}\left(t\right)\left({{\text{K}}_{\text{Bias}}^{\text{b}}}^{\prime }\right)dt & ={\text{C}}_{\text{b}}^{\text{n}}\left(\xi \right)\int_{t_{0}}^{t_{1}}\left({{\text{K}}_{\text{Bias}}^{\text{b}}}^{\prime }\right)dt\\ & ={\text{C}}_{\text{b}}^{\text{n}}\left(\xi \right)\left({{\text{K}}_{\text{Bias}}^{\text{b}}}^{\prime }\right)\left(t_{1}-t_{0}\right)=\left({{\text{K}}_{\text{Bias}}^{\text{n}}}^{\prime }\right)\Delta t \end{array}$$

In Equation (8), there is *ξ* ∈ (*t*_0_, *t*_1_) and ${{\text{K}}_{\text{Bias}}^{\text{n}}}^{\prime }$ in Equation (8) retains the stochastic characteristics of ${{\text{K}}_{\text{Bias}}^{\text{b}}}^{\prime }$.

According to Equations (2), (7) and (8) and the error propagation rule, the VCMs of **γ**^n^ and ${\text{b}}_{\text{I}}^{\text{n}}$ can now be derived:

(9) $$\begin{array}{l} {\text{Q}}_{{\text{γ}}^{\text{n}}}=\sigma_{{\text{γ}}_{\Delta t}^{\text{n}}}^{2}{\text{I}}_{3}+{\text{Q}}_{{\text{γ}}_{0}^{\text{n}}},\\ \sigma_{{\text{γ}}_{\Delta t}^{\text{n}}}^{2}=\sigma_{\text{Bias}}^{2}\Delta t^{2}+\sigma_{\text{Rand}}^{2}\Delta t,\\ {{\text{Q}}_{\text{I}}}^{\prime }=\text{cov}\left({\text{b}}_{\text{I}}^{\text{n}}\right)=\left({\bar{\text{b}}}^{\text{n}}\times \right){\text{Q}}_{{\text{γ}}^{\text{n}}}{\left({\bar{\text{b}}}^{\text{n}}\times \right)}^{\text{T}} \end{array}$$

where Δ*t* = *t*_1_ − *t*_0_, $\sigma_{\text{Bias}}^{2}$ and $\sigma_{\text{Rand}}^{2}$ denote the variances of the components contained in ${\text{K}}_{\text{Bias}}^{\text{b}}$ and ${\text{w}}_{\text{Rand}}^{\text{b}}\cdot {\text{Q}}_{{\text{γ}}_{0}^{\text{n}}}$ is the VCM of the initial attitude measurement provided by the GNSS attitude determination system. Assuming that the GNSS attitude determination system has the equal length but orthogonal dual-baseline style, then ${\text{Q}}_{{\text{γ}}_{0}^{\text{n}}}$ can be written as [20]:

(10) $${\text{Q}}_{{\text{γ}}_{0}^{\text{n}}}=\frac{\sigma_{\Phi }^{2}}{b^{2}}\left\{2{\text{I}}_{3}-{\bar{\text{C}}}_{\text{b}}^{\text{n}}\;\left[\begin{array}{l} 0\\ 0\\ 1 \end{array}\right]\;\left[\begin{array}{ccc} 0 & 0 & 1 \end{array}\right]{\bar{\text{C}}}_{\text{n}}^{\text{b}}\right\}$$

where *b* is the baseline length, $\sigma_{\Phi }^{2}$ denotes the variance of the GNSS carrier phase measurement. Substituting Equation (10) into Equation (9), then we obtain:

(11) $${{\text{Q}}_{\text{I}}}^{\prime }={\bar{\text{C}}}_{\text{b}}^{\text{n}}\;\left[\begin{array}{ccc} 0 & 0 & 0\\ 0 & b^{2}\sigma_{{\text{γ}}_{\Delta t}^{\text{n}}}^{2}+\sigma_{\Phi }^{2} & 0\\ 0 & 0 & b^{2}\sigma_{{\text{γ}}_{\Delta t}^{\text{n}}}^{2}+2\sigma_{\Phi }^{2} \end{array}\right]\;{\bar{\text{C}}}_{\text{n}}^{\text{b}}$$

Equation (11) is derived basing on the presumption of **b̄**^b^ = [*b*, 0, 0]^*T*^. For convenience in the following analysis, **Q**_I_′ can be enlarged to:

(12) $${\text{Q}}_{\text{I}}=\left(b^{2}\sigma_{{\text{γ}}_{\Delta t}^{\text{n}}}^{2}+2\sigma_{\Phi }^{2}\right){\text{I}}_{3}=\sigma_{\text{I}}^{2}{\text{I}}_{3}$$

Comparing with **Q**_I_′, **Q**_I_ evaluates the precision of ${\text{b}}_{\text{I}}^{\text{n}}$ more conservatively.

### 2.2. The Geometry Characteristics of the Inertial Attitude Aided Ambiguity Search Space

The GNSS attitude determination system with low-end receivers usually makes use of the double difference carrier phase measurement model [20], its SF/SE-GNSS counterpart is [24]:

(13) $$\begin{array}{ccc} \text{y}=\lambda {\text{D}}^{T}\cdot \text{ΔΦ}={\text{D}}^{T}\text{Ab}+\lambda \text{a}+\text{n}, & \text{b}\in {\mathbb{R}}^{3}, & \text{a}\in {\mathbb{Z}}^{m-1} \end{array}$$

where the dimensions of **y** and **ΔΦ** are m-1 and m respectively; **D***^T^* is a double difference operator matrix with the dimension (m − 1) × m; **A** is constructed by the Line-Of-Sight (LOS) unit vectors between user and the available satellites; **b** denotes the baseline vector.

The VCM of **y** is given as:

(14) $$\begin{array}{cc} {\text{Q}}_{\text{y}}=\sigma_{\text{Δ}\Phi }^{2}{\text{D}}^{T}\text{D}, & \sigma_{\text{Δ}\Phi }^{2}=2\sigma_{\Phi }^{2} \end{array}$$

The standard deviation of un-differential GNSS carrier phase observation *σ*_Φ_ has the millimeter order of magnitude [25,26].

Substitute ${\text{b}}_{\text{I}}^{\text{n}}$ into Equation (13) and a float solution vector **a** can be resolved:

(15) $$\left\{ \begin{array}{l} {\text{â}}_{\text{I}}=\frac{1}{\lambda }\left(\text{y}-{\text{D}}^{T}\text{A}{\text{b}}_{\text{I}}^{\text{n}}\right),\\ {\text{Q}}_{{\text{â}}_{\text{I}}}=\frac{1}{\lambda^{2}}\left({\text{Q}}_{\text{y}}+{\text{D}}^{T}\text{A}{\text{Q}}_{\text{I}}{\text{A}}^{T}\text{D}\right). \end{array}\right.$$

**â**_I_ is the so-called float solution of Inertial Derived Ambiguity Vector (IDAV).

With **â**_I_ and **Q**_**â**_I__, a double difference ambiguity search space can be written as:

(16) $$T_{\text{I}}\;\left(\text{a}\right)={\left({\text{â}}_{\text{I}}-\text{a}\right)}^{T}{\text{Q}}_{{\text{â}}_{\text{I}}}^{-1}\left({\text{â}}_{\text{I}}-\text{a}\right)\leq \chi^{2}$$

The ambiguity search space described by Equation (16) is a (hyper)ellipsoid. Both its center and shape vary with the reference satellite selection. Therefore, before analyzing the geometry characteristics of T_I_ (**a**) ≤ *χ*^2^, it is necessary to lift the standard double difference grid **a** ∈ ℤ*^m^*^−1^ into the single difference ambiguity search space ℝ*^m^* [27], then an ambiguity search space that is independent of the selection of reference satellite can be obtained:

(17) $$\begin{array}{l} {\text{T}}_{\text{I}}\;\left({\text{s}}^{\prime }\right)={\left({\text{ŝ}}_{\text{I}}^{\prime }-{\text{s}}^{\prime }\right)}^{T}\text{D}{\text{Q}}_{{\text{â}}_{\text{I}}}^{-1}{\text{D}}^{T}\left({\text{ŝ}}_{\text{I}}^{\prime }-{\text{s}}^{\prime }\right)\leq \chi^{2},\\ {\text{â}}_{\text{I}}={\text{D}}^{T}{\text{ŝ}}_{\text{I}}^{\prime },\;\text{a}={\text{D}}^{T}{\text{s}}^{\prime }. \end{array}$$

As the equivalent form of Equations (16) and (17) can be regarded as the standard form of the inertial attitude aided ambiguity search space.

The shape of the (hyper)ellipsoid T_I_ (**s′**) ≤ *χ*^2^ is mainly determined by $\text{D}{\text{Q}}_{{\text{â}}_{\text{I}}}^{-1}{\text{D}}^{T}$. Referring to the proof of theorem 1 in [27], if *m* is larger than 4, the eigenvalue decomposition of $\text{D}{\text{Q}}_{{\text{â}}_{\text{I}}}^{-1}{\text{D}}^{T}$ is given as:

(18) $$\begin{array}{l} \text{D}{\text{Q}}_{{\text{â}}_{\text{I}}}^{-1}{\text{D}}^{T}=\text{R}\Lambda {\text{R}}^{T},\\ \text{Λ}=\text{diag}\left(0,\frac{\lambda^{2}}{2\sigma_{\Phi }^{2}}{\text{I}}_{m-4},\frac{\lambda^{2}}{2\sigma_{\Phi }^{2}}{\left({\text{I}}_{3}+\text{Γ}\right)}^{-1}\right),\\ \text{R}=\left[\begin{array}{ccc} \text{w} & \text{V} & \text{U} \end{array}\right]. \end{array}$$

where:

(19) $$\begin{array}{l} \text{Γ}=\text{diag}\left[\begin{array}{ccc} \gamma_{1}\left({\text{s}}_{1}\right) & \gamma_{2}\left({\text{s}}_{2}\right) & \gamma_{3}\left({\text{s}}_{3}\right) \end{array}\right],\\ \begin{array}{cc} \text{w}={\text{e}}_{m}{\left({\text{e}}_{m}^{T}{\text{e}}_{m}\right)}^{-1/2}, & \text{V}=\text{E}{\left({\text{E}}^{T}\text{E}\right)}^{-1/2}, \end{array}\\ \begin{array}{ccc} \text{U}=\text{F}{\left({\text{F}}^{T}\text{F}\right)}^{1/2}, & \text{F}={\text{PAS}}^{-T}, & \text{S}=\left[\begin{array}{ccc} {\text{s}}_{1} & {\text{s}}_{2} & {\text{s}}_{3} \end{array}\right] \end{array}. \end{array}$$

In Equation (19), **e***_m_* is the m-dimension vector with all elements equal to 1; **E** is a basis matrix of R(**D**) ∩ N(**A***^T^***P**), where N(**A***^T^***P**) describes the null space of **A***^T^***P** and R(**D**) means the column space of **D**; **P** = **D**(**D***^T^***D**)^−1^**D***^T^* is the orthogonal projection operator of R(**D**); The baseline gain *γ_i_*(**s***_i_*), *i*=1,2,3 is defined as:

(20) $$\begin{array}{cc} \gamma_{i}\left({\text{s}}_{i}\right)=\frac{{\text{s}}_{i}^{T}{\text{Q}}_{\text{I}}{\text{s}}_{i}}{{\text{s}}_{i}^{T}{\text{Q}}_{\overset{⌣}{\text{b}}}\left(\Phi \right){\text{s}}_{i}}, & i=1,2,3 \end{array}$$

Different from the baseline gain number defined in [24], the gain number defined by Equation (20) describes that in the direction of gain vector **s***_i_* ∈ ℝ^3^, the accuracy of the baseline solution **b**⌣(Φ) varies with respect to ${\text{b}}_{\text{I}}^{\text{n}}\cdot {\text{Q}}_{\overset{⌣}{\text{b}}}$ is the VCM of **b**⌣(Φ). It can be proved that if **PA** is full column rank, **s***_i_* will be the unit eigenvector for the eigenvalue decomposition of **A***^T^***PA**= (**PA**)*^T^*·(**PA**), which means:

(21) $$\begin{array}{l} {\text{A}}^{T}\text{PA}=\text{S}\Sigma {\text{S}}^{T},\\ \text{S}=\left[\begin{array}{ccc} {\text{s}}_{1} & {\text{s}}_{2} & {\text{s}}_{3} \end{array}\right],\\ \Sigma =\text{diag}\left(\xi_{1},\xi_{2},\xi_{3}\right) \end{array}$$

Since **A***^T^***PA** is positive definitive, the three eigenvalues in Equation (21) satisfy that *ξ_i_* > 0, *i* = 1,2,3,. Then, it can be proved that the baseline gain *γ_i_* (**s***_i_*), *i* = 1,2,3 are the functions of *ξ_i_* > 0, *i* = 1,2,3:

(22) $$\begin{array}{cc} \gamma_{i}\left({\text{s}}_{i}\right)=\frac{\sigma_{\text{I}}^{2}}{2\sigma_{\Phi }^{2}}\xi_{i}, & i=1,2,3 \end{array}$$

Hence, with Equation (17) in Equation (22), the decoupled expansion of the inertial attitude aided ambiguity search space can be written as:

(23) $$T_{\text{I}}\;\left({\text{s}}^{\prime }\right)=\frac{\lambda^{2}}{2\sigma_{\Phi }^{2}}\left\{\sum_{i=1}^{3}\frac{1}{\left(\gamma_{i}+1\right)}{\left[{\text{u}}_{i}^{T}\left({\text{ŝ}}_{\text{I}}^{\prime }-{\text{s}}^{\prime }\right)\right]}^{2}+\sum_{i=1}^{m-4}{\left[{\text{v}}_{i}^{T}\left({\text{ŝ}}_{\text{I}}^{\prime }-{\text{s}}^{\prime }\right)\right]}^{2}\right\}\leq \chi^{2}$$

From Equation (23), it is obvious that the principal axis lengths in the V-axis of the (hyper)ellipsoid T_I_ (**s′**) ≤ *χ*^2^ are all equal to $\sqrt{2}\chi \sigma_{\Phi }/\lambda$, and the three principal axis lengths in U-axis are equal to:

(24) $$lu_{i}=\sqrt{\gamma_{i}+1}\cdot \left(\frac{\sqrt{2}\chi \sigma_{\Phi }}{\lambda }\right)\begin{array}{cc} =\frac{\chi \sigma_{\Phi }}{\lambda }\sqrt{{\text{Ratio\_IG}}^{2}\cdot \xi_{i}+2}, & i=1,2,3 \end{array}$$

In Equation (24), *Ratio*_*IG* = *σ*_1_/*σ*_Φ_; *ξ_i_*, *i* = 1,2,3 are not only the eigenvalues of **A***^T^***PA**, but also the singular values of **PA**. **PA** can be seen as the projection of the user-satellite geometry matrix **A** onto R(**D**). Thus, *ξ_i_*, *i* = 1,2,3 describe the geometry of the used satellite.

## 3. Analysis for Augmenting the SF/SE-GNSS Model Strength with Inertial Attitude Aided

From the double-difference measurement model of the SF/SE-GNSS compass, the ambiguity float solution vector and its VCM can be resolved:

(25) $$\left\{ \begin{array}{l} \text{â}=\frac{1}{\lambda }\left[\text{y}-{\text{D}}^{T}\text{A}\hat{\text{b}}\left(\Phi ,\rho \right)\right],\\ {\text{Q}}_{\text{â}}=\frac{1}{\lambda^{2}}\left[2\sigma_{\Phi }^{2}{\text{D}}^{T}\text{D}+{\text{D}}^{T}\text{A}{\text{Q}}_{\hat{\text{b}}}\left(\rho \right){\text{A}}^{T}\text{D}\right] \end{array}\right.$$

In Equation (25), both **b̂**(Φ, *ρ*) and **â** are the real-valued estimations of the double-difference measurement model of the SF/SE-GNSS compass, where (Φ, *ρ*) shows that the baseline vector estimation **b̂** uses both code and phase observations. The necessary condition for Equation (25) is the unique solution is that the number of available satellites is more than 4. The standard form of the ambiguity search space determined by **â** and **Q_â_** is:

(26) $$T\left({\text{s}}^{\prime }\right)={\left({\text{ŝ}}^{\prime }-{\text{s}}^{\prime }\right)}^{T}\text{D}{\text{Q}}_{\text{â}}^{-1}{\text{D}}^{T}\left({\text{ŝ}}^{\prime }-{\text{s}}^{\prime }\right)\leq \chi^{2}$$

Comparing Equation (17) and Equation (26), it can be proved that $\text{D}{\text{Q}}_{\text{â}}^{-1}{\text{D}}^{T}$ has the same eigenvectors as those of $\text{D}{\text{Q}}_{{\text{â}}_{\text{I}}}^{-1}{\text{D}}^{T}$, *i.e.*, **w**, **V** and **U**; the eigenvalues corresponding to **w** and **V** are equal to those in the $\text{D}{\text{Q}}_{{\text{â}}_{\text{I}}}^{-1}{\text{D}}^{T}$ case, respectively; the eigenvalues of **U** = (**u**_1_,**u**_2_,**u**_3_) are equal to $\lambda^{2}/2\left(\sigma_{\rho }^{2}+\sigma_{\Phi }^{2}\right)$ [27], or the axis lengths in the directions of **U**-axis of (hyper)-ellipsoid T (**s′**) ≤ *χ*^2^ are all equal to:

(27) $$lu^{\prime }=\frac{\chi \sigma_{\Phi }}{\lambda }\sqrt{{\text{Ratio\_PC}}^{2}\cdot 2+2}$$

where *Ratio*_*PC* = *σ_ρ_*/*σ*_Φ_, *σ_ρ_* is the standard deviation of code observation.

Comparing Equation (27) with Equation (24), the variations caused by inertial attitude measurement will be reflected in the directions of **U**-axis, and *lu′* is independent of the user-satellite geometry, which is revealed by *ξ_i_*=2 in Equation (27). If *Ratio*_*IG*^2^·*ξ_i_* < *Ratio*_*PC*^2^·2, or $\sigma_{\text{I}}^{2}<\sigma_{\rho }^{2}\cdot \left(2/\xi_{i}\right),i=1,2,3$, there will be *lu_i_* < *lu′*, thus it means that the volume of T_I_ (**s′**) ≤ *χ*^2^ is smaller than that of T (**s′**) ≤ *χ*^2^, or the inertial attitude measurement decreases the unaided SF/SE-GNSS ambiguity search space.

**Figure 1.**
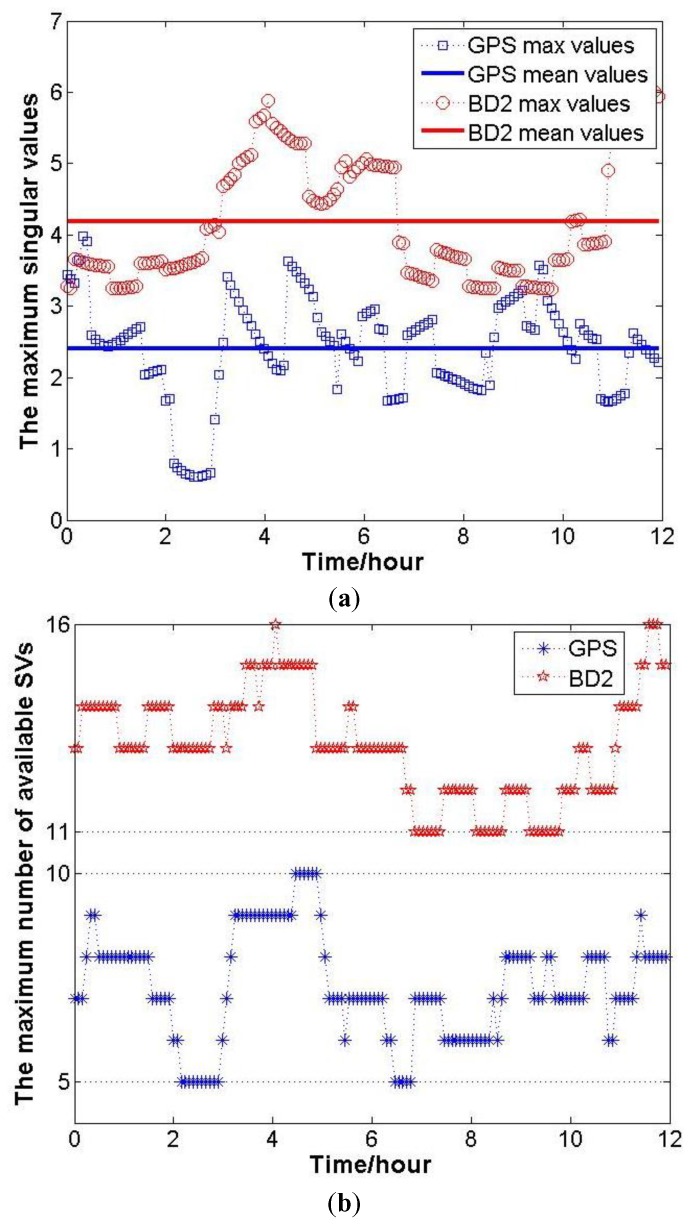
(**a**) The variation of *ξ*_max_ and the mean values during 12 h in Changsha; (**b**) The variation of maximum numbers of available satellites in Changsha during 12 h.

The magnitude of *ξ_i_* can be revealed by the simulation results. Firstly, based on the GNSS Yuma file, the positions of GPS and BD2 satellites within 12 h can be calculated. Then, setting the user’s position on Earth, the maximum number of available satellites can be determined, and the geometry matrix **A** is constructed. Finally, *ξ_i_*, *i* = 1,2,3 can be obtained by the eigenvalue decomposition of **A***^T^***PA** at each epoch. In this simulation, the user position is set as one point in Changsha, 28.2202°N, 112.9925°E, and the number of available satellites and **A** are recorded every 6 min. At each recording epoch *k*, the maximum eigenvalue of **A***^T^***PA** is denoted as *ξ_i_*(*k*), *i* − 1,2,3. The variation of *ξ*_max_ during 12 h and the mean values are shown in Figure 1a.

It must be noted that to calculate *ξ*_max_ at each recording epoch, all the available satellites are used. Hence, the corresponding maximum numbers of available satellites are shown in Figure 1b. Above all, for the inertial attitude measurement decreasing the SF/SE-GNSS ambiguity search space, a sufficient condition is that:

(28) $$\sigma_{\text{I}}^{2}<\frac{2}{\max\left(\xi_{\max}\right)}\sigma_{\rho }^{2}$$

Equation (28) can be used as an empirical formula for selecting the inertial sensors for inertial attitude augmentation. Until now, the analytical relation between the inertial attitude measurement and the SF/SE-GNSS ambiguity search space has been built, and the influences of the inertial attitude measurement to the unaided SF/SE-GNSS ambiguity search space are directly revealed. From the perspective of geometry, the mechanism of inertial attitude augmentation is revealed for the first time.

To describe the model strength of SF/SE-GNSS ambiguity resolution, the ADOP is introduced. This subsection will quantitatively describe the augmentation effect based on inertial attitude measurement with different ADOPs. The formula defining ADOP is given by [28]:

(29) $$\text{ADOP}=\sqrt[{1/m-1}]{\text{det}\left({\text{Q}}_{\text{â}}\right)}$$

where m − 1 is the dimension of **Q_â_**. ADOP has the same unit as ambiguity, *i.e.*, cycle.

According to Equation (29), the determinant of VCM of the ambiguity float solution vector is needed to calculate ADOP. Based on Equation (15), the determinant of **Q_â_**_I_ equals to:

(30) $$\left|{\text{Q}}_{{\text{â}}_{\text{I}}}\right|={\left(\frac{2\sigma_{\Phi }^{2}}{\lambda^{2}}\right)}^{m-1}\left|{\text{D}}^{T}\text{D}+\frac{1}{2\sigma_{\Phi }^{2}}{\text{D}}^{T}\text{A}{\text{Q}}_{\text{I}}{\text{A}}^{T}\text{D}\right|$$

Utilizing the determinant decomposition formula [29]:

(31) $$\left|\text{E}-\text{F}{\text{G}}^{-1}\text{H}\right|\left|\text{G}\right|=\left|\text{E}\right|\left|\text{G}-\text{H}{\text{E}}^{-1}\text{F}\right|$$

Together with Equation (31), Equation (30) can be rewritten as:

(32) $$\left|{\text{Q}}_{{\text{â}}_{\text{I}}}\right|={\left(\frac{2\sigma_{\Phi }^{2}}{\lambda^{2}}\right)}^{m-1}\left|{\text{D}}^{T}\text{D}\right|\left|{\text{I}}_{3}+{\text{Q}}_{\text{I}}{\text{Q}}_{\overset{⌣}{\text{b}}}^{-1}\left(\Phi \right)\right|$$

In Equation (32), it is easy to obtain that **|D***^T^***D|**=*m*. According to Equations (12)–(14), it can be proved that ${\text{Q}}_{\text{I}}{\text{Q}}_{\overset{⌣}{\text{b}}}^{-1}\left(\Phi \right)=\text{S}\Gamma {\text{S}}^{T}$, thus:

(33) $$\left|{\text{Q}}_{{\text{â}}_{\text{I}}}\right|=m\cdot {\left(\frac{2\sigma_{\Phi }^{2}}{\lambda^{2}}\right)}^{m-1}\overset{3}{\underset{i=1}{\text{∏}}}\left(1+\frac{1}{2}\cdot \text{Ratio\_IG}\cdot \xi_{i}\right)$$

The same process can be imposed on Equation (25). Then the determinant of **Q_â_** equals:

(34) $$\left|{\text{Q}}_{\text{â}}\right|=m\cdot {\left(\frac{2\sigma_{\Phi }^{2}}{\lambda^{2}}\right)}^{m-1}{\left(1+\frac{1}{2}\cdot \text{Ratio\_PC}\cdot 2\right)}^{3}$$

Substituting Equations (33) and (34) into Equation (29):

(35) $$\begin{array}{l} {\text{ADOP}}_{\text{I}}={\sqrt{\left|{\text{Q}}_{{\text{â}}_{\text{I}}}\right|}}^{\frac{1}{m-1}}=\frac{2\sigma_{\Phi }^{2}}{\lambda^{2}}\cdot {\sqrt{m\cdot \overset{3}{\underset{i=1}{\text{∏}}}\left(1+\frac{1}{2}\cdot {\text{Ratio\_IG}}^{2}\cdot \xi_{i}\right)}}^{\frac{1}{m-1}},\\ {\text{ADOP}}_{\text{SF}/\text{SE}}={\sqrt{\left|{\text{Q}}_{\text{â}}\right|}}^{\frac{1}{m-1}}=\frac{2\sigma_{\Phi }^{2}}{\lambda^{2}}\cdot {\sqrt{m\cdot {\left(1+\frac{1}{2}\cdot {\text{Ratio\_PC}}^{2}\cdot 2\right)}^{3}}}^{\frac{1}{m-1}} \end{array}$$

In Equation (35), *ADOP*_I_ and *ADOP*_SF/SE_ represent the ADOPs of the inertial attitude augmentation model and unaided SF/SE-GNSS model, respectively. According to Equation (35), it is known that, if a certain accuracy level of GNSS measurement was given, *ADOP*_SF/SE_ would only be determined by the number of available satellites, and the user-satellite geometry has no effect on *ADOP*_SF/SE_. Besides this, it also can be derived that Equation (28) is a sufficient condition for augmenting the SF/SE-GNSS model strength with the aided inertial attitude.

**Figure 2.**
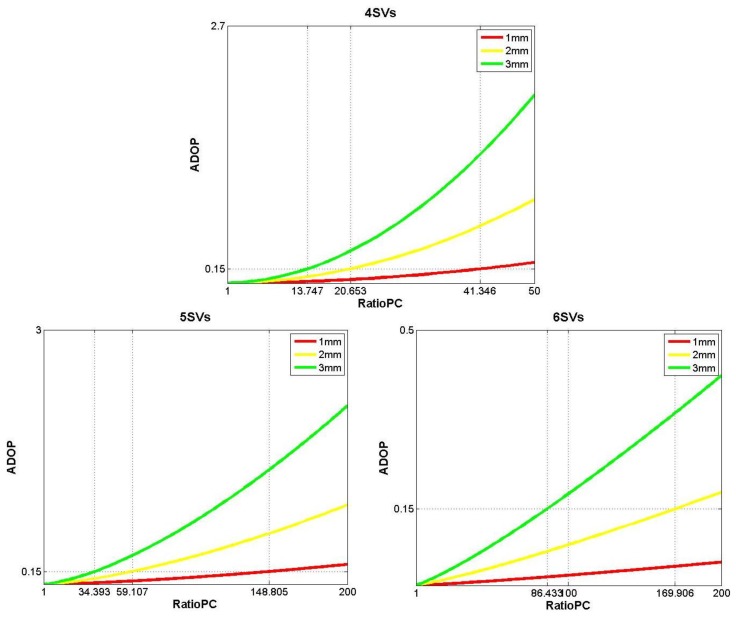
The variation of *ADOP*_SF/SE_ when the number of available satellites equals four, five and six, respectively.

Considering that the number of available satellites equals four, five and six, the simulation results indicating how the *ADOP*_SF/SE_ values vary with respect to *σ*_Φ_ and *Ratio*_*PC*, are explicitly shown in Figure 2.

In Figure 2, the varying ranges of *σ*_Φ_ and *Ratio*_*PC* are both determined by the actual performance of several GNSS receivers, which are listed in Tables 1–3.

This paper will implement the ambiguity resolution basing on the integer aperture estimator with fixed-failure rate [30,31]. This estimator requires that the success rate of ambiguity resolution be larger than 0.99, which corresponds to the interval *ADOP*≤0.15. Hence, the reference level of ADOP appeared in Figure 2 was set as 0.15.

The simulation results shown in the first (upper) subplot of Figure 2 indicate that when *ADOP*_SF/SE_≤0.15, while there are four available satellites, the values of *Ratio*_*PC* cannot be achieved by current GNSS receivers. If the number of available satellites is five and *σ*_Φ_ ≥ 2 mm, *i.e.*, the green line and the yellow line shown in the second (bottom, left) subplot of Figure 2, the values of *Ratio*_*PC* make *ADOP*_SF/SE_≤0.15 are still hard to satisfy with current low-end GNSS receivers. As to six available satellites and *σ*_Φ_≤1 mm, the *ADOP*_SF/SE_ values in the given interval of *Ratio*_*PC* will always be less than 0.15, just like the results shown by the red line in the third (bottom, right) subplot of Figure 2.

To weaken the model strength of the SF/SE-GNSS compass with aided inertial attitude measurement, in the following simulation, *σ*_Φ_ is set to be 3 mm and let *ξ_i_* be equal to 3. The precision combinations for generating the MEMS gyro measurements are shown in Table 4.

The MEMS accelerator bias and noise level are chosen as 5×10^−3^ g/h and 1×10^−3^ g/h, respectively. Since only the extra-short baseline scenario is considered, the baseline length is set to be successively 0.5, 1.0, 1.5 and 2.0 m.

**Figure 3.**
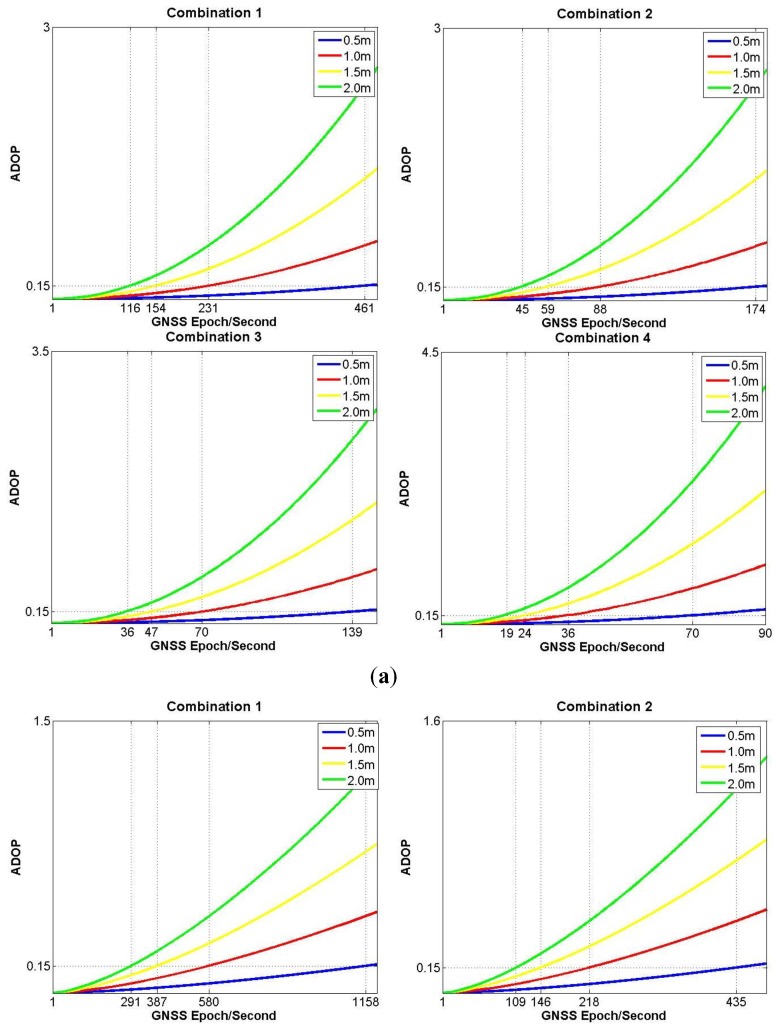

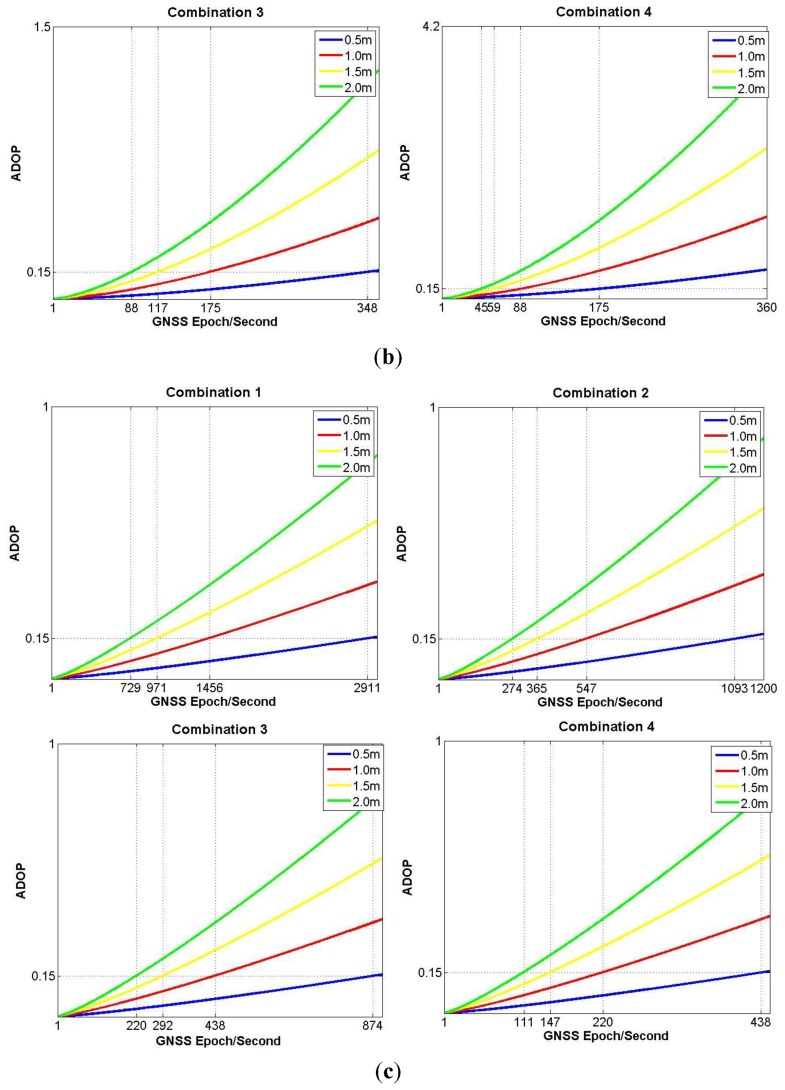
(**a**) The variation of *ADOP*_I_ with four satellites available; (**b**) The variation of *ADOP*_I_ with five satellites available; (**c**) The variation of *ADOP*_I_ with six satellites available.

When the number of available satellites successively equals four, five and six (Figure 3), the simulation results for *ADOP*_I_ are generated under different conditions of baseline lengths and MEMS gyro precision combinations.

In Figure 3a, where only four satellites are available, if the worst accuracy combination 4 is chosen, the state of *ADOP*_I_≤0.15 can only be kept about 1 min. Otherwise, if the best accuracy combination 1 is used, the time span for *ADOP*_I_≤0.15 is still no more than 2 min. Hence, Figure 3a demonstrates that four available satellites will be a very weak condition for the model strength of SF/SE-GNSS compass with aided inertial attitude measurement. In Figure 3b, where five satellites are available and the baseline length equals to 1.5 or 2.0 m, the worst accuracy combination 4, or the second best accuracy combination 2 still cannot keep *ADOP*_I_≤0.15 for more than 3 min. In Figure 3c, whatever the accuracy combination is chosen, the MEMS-IMU attitude augmentation can already keep *ADOP*_I_≤0.15 more than 3 min, meaning the SF/SE-GNSS model with aided inertial attitude has some strength when six available satellites are considered.

Combining the simulation results shown in Figures 2 and 3, the number of available satellites is the principal factor that influences the SF/SE-GNSS model strength. Based on the current accuracy levels of the low-end GNSS receivers and MEMS gyros, the key to realize the inertial attitude augmentation for SF/SE-GNSS ambiguity resolution is to resolve the existing problems when the number of available satellites is between four and six.

## 4. Performance Analysis for the SF/SE-GNSS Ambiguity Resolution with Aided Inertial Attitude

In this section, a specific simulation experiment will be designed with the low-end GNSS receivers and the MEMS-IMU considered. In the framework of an integer aperture estimator with fixed-failure rate, the performance of SF/SE-GNSS ambiguity with aided inertial attitude will be analyzed based on the simulation results, while the rate ratio test and the difference test will be compared under different model strength scenarios.

### 4.1. Simulation Experiment Design

**Figure 4.**
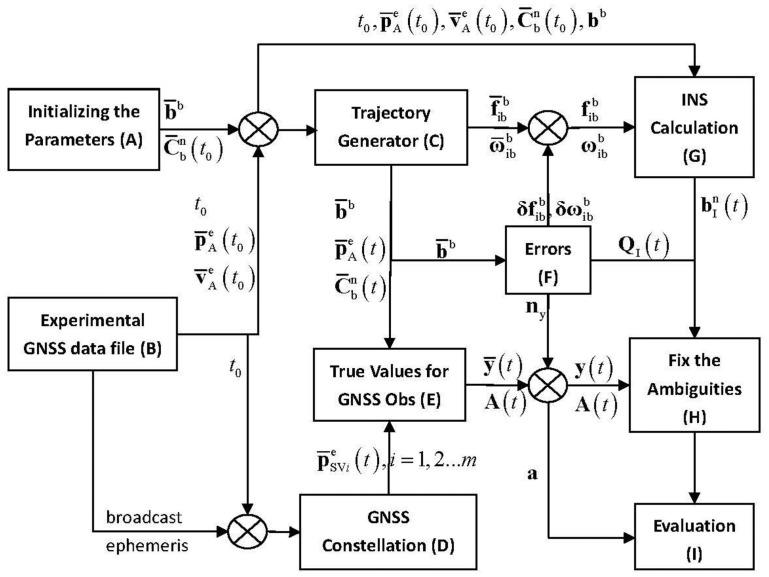
Simulation flow chart of inertial attitude augmentation for SF/SE-GNSS ambiguity resolution.

Figure 4 explicitly describes the basic simulation flow chart.

The main functions of each module in Figure 4 are introduced here:

(1) Module A. Assume that the geometric center of primary antenna A and the origin of b-frame overlap each other, the true baseline vector in b-frame and initial vehicle attitude are known in prior, which are labeled as **b̄**^b^ and ${\bar{\text{C}}}_{\text{b}}^{\text{n}}\left(t_{0}\right)$ respectively.
(2) Module B. As to the GNSS data collected from an field experiment, the GNSS position and velocity measurements at certain epoch are used as the true initial navigation states, which include the time, position and velocity for INS calculation. These parameters are denoted as *t*_0_, ${\bar{\text{p}}}_{\text{A}}^{\text{e}}\left(t_{0}\right)$ and ${\bar{\text{v}}}_{\text{A}}^{\text{e}}\left(t_{0}\right)$, successively.
(3) Module C. The trajectory generator outputs the true values of vehicle position, velocity, attitude and IMU measurements, which are denoted as ${\bar{\text{p}}}_{\text{A}}^{\text{e}}\left(t\right)$, ${\bar{\text{v}}}_{\text{A}}^{\text{e}}\left(t\right)$, ${\bar{\text{C}}}_{\text{b}}^{\text{n}}\left(t\right)$, ${\bar{\text{f}}}_{\text{ib}}^{\text{b}}$ and ${\bar{\text{ω}}}_{\text{ib}}^{\text{b}}$, and there is *t*≥*t*_0_.
(4) Module D. The true satellite positions are calculated from the broadcast ephemeris, which is also collected in the same field experiment of Module B, and the true satellite positions are labeled as ${\bar{\text{p}}}_{\text{SV}i}^{\text{e}}\left(t\right),i=1,2\ldots m$.
(5) Module E. With the true navigation states utilized, the true baseline vector, satellite positions, un-differenced code and carrier phase observations are generated, which are labeled and included in **ȳ**(*t*). The true ambiguities are included in the vector **a**, and the user-satellite unit LOS vectors form the design matrix **A**(*t*).
(6) Module F. After adding the simulation noises into the original outputs of Module C and Module E respectively, the MEMS-IMU and GNSS measurements can be obtained and denoted as ${\text{f}}_{\text{ib}}^{\text{b}}$, ${\text{ω}}_{\text{ib}}^{\text{b}}$ and **y**(*t*). According to the standard deviations of the added noises and the setting baseline length, the VCM of IDBV can be calculated.
(7) Module G. Using the MEMS-IMU measurements ${\text{f}}_{\text{ib}}^{\text{b}}$ and ${\text{ω}}_{\text{ib}}^{\text{b}}$, the SINS calculation starts from the initial navigation states. Besides the SINS navigation states, this module also outputs the IDBV ${\text{b}}_{\text{I}}^{\text{n}}\left(t\right)$.
(8) Module H. Basing on the SF/SE-GNSS float ambiguity vectors given in Equations (15) and (25), the integer aperture estimator with fixed failure rate tries to fix the ambiguities.
(9) Module I. This module compares the ambiguity solutions output from module H with the true ambiguity **a**, and evaluates the performance of SF/SE-GNSS ambiguity resolution with inertial attitude measurements aided.

It should be noted that the influence of the user position error on **A**(*t*) can be omitted [32], hence in the transmission process from module E to H, module F doesn't add any measurement error to **A**(*t*). Considering the double difference operation to $\Phi_{\text{j}}^{\text{SV}i}\left(t\right)$ in module H, module F does not model the propagation errors and clock errors contained in the original GNSS observation vector **y**(*t*). Hence, the noise item **n**_y_ can be modeled as a white noise vector. Otherwise, in module F, the modeling methods of MEMS-IMU angular velocity measurement error are the same as those for $\text{δ}{\text{ω}}_{\text{ib}}^{\text{b}}$ in Equation (6).

**Figure 5.**
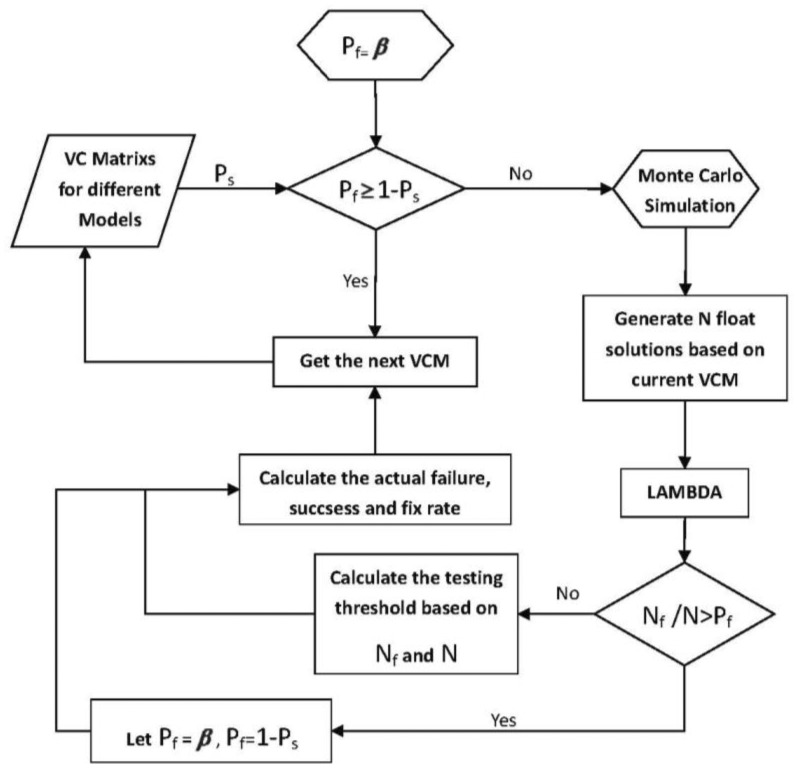
Determination of the testing threshold in the framework of integer aperture estimator with fixed failure rate.

The integer aperture estimator with fixed failure rate can control its failure rate by setting the testing threshold. However, there is no analytical relation between the testing threshold and the failure rate. Herein, the threshold will be determined by Monte Carlo simulation. Figure 5 gives the detailed steps for the determination of testing threshold in the framework of integer aperture estimator with fixed failure rate. In Figure 5, *N*_f_ denotes the number of failing samples in ambiguity resolution. The number of simulations is set as 100,000.

### 4.2. Performance Analysis

Since the approximation Equation (4) omits the influence of the vehicle dynamics, the simulation in this subsection will only consider the scenario of a static vehicle and be carried out three times based on the number of available satellites. The performance analysis for SF/SE-GNSS ambiguity resolution also focuses on the influence of model strength varying from weak to strong, *i.e.*, *ADOP*_I_ ranges from 0.1 to 0.5. In the framework of an integer aperture estimator with fixed failure rate, the ratio test and difference test will be implemented separately. To evaluate the simulation results, the ambiguity solutions are classified and summarized in Table 5.

**Figure 6.**
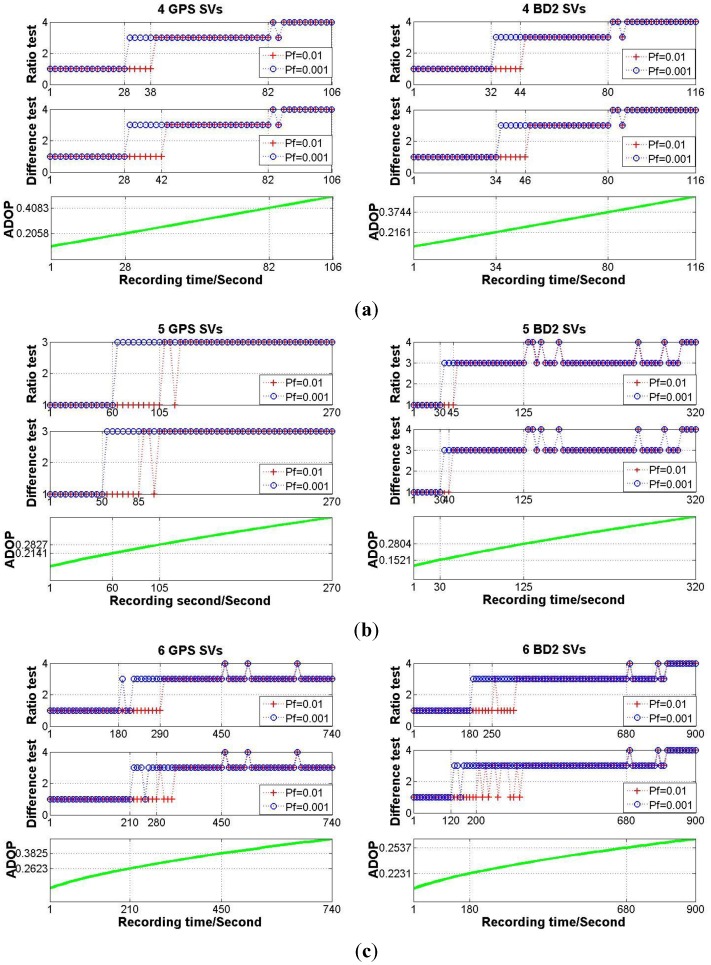
(**a**) The MEMS-IMU attitude aiding SF/SE-GNSS ambiguity resolution results with four satellites available; (**b**) The MEMS-IMU attitude aiding SF/SE-GNSS ambiguity resolution results with five satellites available; (**c**) The MEMS-IMU attitude aiding SF/SE-GNSS ambiguity resolution results with six satellites available.

According to the simulation flow chart shown in Figure 4, each simulation experiment period is set as 1000 s. The baseline length is set as 2 m. The accuracy combination 2 shown in Table 4 is chosen for the MEMS gyros, and let *σ*_Φ_ equal to 0.003 m. Based on the broadcast ephemera collected in the field experiment, the GPS or BD2 constellation is generated, and the numbers of available satellites for GPS and BD2 is equal to eight and 11, respectively. Under any one of the conditions that the number of available satellites is limited to be four, five or six, the satellite combination which gives the weakest model strength, or in other words, the maximum *ADOP*_I_ value, is picked out from all the available satellites. The fixed failure rate for integer aperture estimator is set as 0.01 and 0.001. For different simulation scenarios, the SF/SE-GNSS ambiguity resolution results with MEMS-IMU attitude aided will be given in Figure 6.

From Figure 6, the output results of integer aperture estimator varied from 1 to 3 or 4, Style 2 never appeared in the process for model strength varying from strong to weak. It reveals that when the MEMS-IMU attitude measurements are used to augment the SF/SE-GNSS model strength, the integer aperture estimator with fixed failure rate has good reliability once the testing threshold is determined. Even if the requirement for failure rate was relaxed, the performance of the SF/SE-GNSS ambiguity resolution still held at a high level. On the other hand, no matter which testing method was selected between the ratio test and the difference test, there was no significant distinction in performance, this means that under the condition of SF/SE-GNSS model with aided inertial attitude, the testing method is not the primary factor that affects the performance of ambiguity resolution.

## 5. Conclusions

This paper was set up based on the GNSS compass model and the MEMS-IMU attitude measurement application. It investigated how to utilize the inertial attitude measurement to enhance the performance of unaided SF/SE-GNSS ambiguity resolution. According to the definition of IDBV, the relationship between the inertial attitude measurement and the ambiguity search space was established. Then, the essence of inertial attitude augmenting the SF/SE-GNSS model strength was revealed from the geometrical perspective, while the empirical formula of selecting the inertial sensors for inertial attitude augmenting was given. ADOP was introduced to quantitatively describe the model strength. Based on the simulation results, the factors influencing SF/SE-GNSS model strength were analyzed overall. It was concluded that among the inertial sensor measurement accuracy, the GNSS measurement accuracy, the baseline length and the number of available satellites, the last factor is the most significant one in practice. In the designed simulation experiment for the SF/SE-GNSS ambiguity resolution with MEMS-IMU aided attitude, the integer aperture estimator with fixed failure rate was used. During the model strength varying from strong to weak, the performance of MEMS-IMU attitude augmenting SF/SE-GNSS ambiguity resolution was analyzed, and some practical suggestions about the application of the integer aperture estimator were given.

## Figures and Tables

**Table 1.** The nominal measurement precisions of three GNSS OEM cards.

	**Frequency**	**Code** (1*σ*)	**Carrier Phase** (1*σ*)	*Ratio*_*PC*
**Novatel OEM628**	GPS-L1	4 cm	0.5 mm	80
**Novatel OEMStar**	GPS-L1	5 cm	0.6 mm	83.3
**UB240**	GPS-L1/BD2-B1	10 cm	0.5 mm	200

**Table 2.** Standard deviation of BD2 code and phase observations in static experiments.

	**C07 (Reference SV)**	**C11**	**C14**	**C04**	**C01**
*σ*_Φ_ (mm)	N/A	1.2864	1.0368	1.1712	0.8448
*σ_ρ_* (cm)	N/A	20.04	13.3	16.29	11.21
*Ratio*_*PC*	N/A	155.8	128.3	139.1	132.7
	**C03**	**C06**	**C02**	**C05**	**C09**
*σ*_Φ_ (mm)	0.8064	0.7872	1.1904	0.8064	1.0176
*σ_ρ_* (cm)	10.78	10.55	14.64	10.66	12.77
*Ratio*_*PC*	133.7	134	123	132.2	125.5

**Table 3.** Standard deviation of GPS code and phase observations in static experiments.

	**G24 (Reference SV)**	**G15**	**G18**	**G14**	**G22**	**G21**
*σ*_Φ_ (mm)	N/A	0.8373	0.5899	1.2369	1.1037	0.7231
*σ_ρ_* (cm)	N/A	9.67	9.5	18.66	13.53	9.68
*Ratio*_*PC*	N/A	115.5	161	150.9	122.6	133.9

**Table 4.** The simulation parameters of MEMS gyro.

	**Gyro Bias** (1*σ*)	**Gyro Noise**(1*σ*)
**Combination 1**	30°/h	$10^{\circ }/\sqrt{\text{h}}$
**Combination 2**	80°/h	$30^{\circ }/\sqrt{\text{h}}$
**Combination 3**	100°/h	$50^{\circ }/\sqrt{\text{h}}$
**Combination 4**	200°/h	$80^{\circ }/\sqrt{\text{h}}$

**Table 5.** Ambiguity resolution results classification.

**Style 1**	Accept correct integer ambiguity
**Style 2**	Accept wrong integer ambiguity
**Style 3**	Reject correct integer ambiguity
**Style 4**	Reject wrong integer ambiguity
